# Postoperative Pain after Root Canal Treatment: A Prospective Cohort Study

**DOI:** 10.1155/2012/310467

**Published:** 2012-03-15

**Authors:** M. Gotler, B. Bar-Gil, M. Ashkenazi

**Affiliations:** ^1^Department of Pediatric Dentistry, The Maurice and Gabriela Goldschleger School of Dental Medicine, Tel Aviv University, 69978 Tel-Aviv, Israel; ^2^Departments of Oral Pathology and Oral Medicine, The Maurice and Gabriela Goldschleger School of Dental Medicine, Tel Aviv University, 69978 Tel Aviv, Israel

## Abstract

*Aim*. To evaluate the incidence and severity of postendodontic treatment pain (PEP) subsequent to root canal treatment (RCT) in vital and necrotic pulps and after retreatment. *Methodology*. A prospective study. Participants were all patients (*n* = 274) who underwent RCT in teeth with vital pulp, necrotic pulp, or vital pulp that had been treated for symptomatic irreversible pulpitis or who received root canal retreatment, by one clinician, during an eight-month period. Exclusion criteria were swelling, purulence, and antibiotic use during initial treatment. A structured questionnaire accessed age, gender, tooth location, and pulpal diagnosis. Within 24 h of treatment, patients were asked to grade their pain at 6 and 18 hours posttreatment, using a 1–5 point scale. *Results*. RCT of teeth with vital pulp induced a significantly higher incidence and severity of PEP (63.8%; 2.46 ± 1.4, resp.) than RCT of teeth with necrotic pulp (38.5%; 1.78 ± 1.2, resp.) or of retreated teeth (48.8%; 1.89 ± 1.1, resp.). No statistical relation was found between type of pain (spontaneous or stimulated) and pulp condition. *Conclusion*. RCT of teeth with vital pulp induced a significantly higher incidence and intensity of PEP compared to teeth with necrotic pulp or retreated teeth.

## 1. Introduction

Prevention and management of postendodontic pain (PEP) is an integral part of endodontic treatment. Informing patients about expected postendodontic pain (PEP) and prescribing medications to manage it can increase patient confidence in their dentists, increase patients' pain threshold, and improve their attitude toward future dental treatment [1, 2]. According to previously published data, pulp therapy and root canal treatment (RCT) induce more frequent and more severe postoperative pain than do other dental operative procedures [3, 4]. In the literature, reported frequencies of PEP range from 1.5 [5] to 53% [3]. The large range is apparently due, in large part, to differences in definitions of postendodontic pain. Most studies that investigated the prevalence of postendodontic pain referred to flare-up, which was defined as severe pain and/or swelling after endodontic treatment, requiring an unscheduled appointment and active treatment. Therefore, patients who experienced pain after endodontic treatment and did not require active treatment were excluded from those studies [6].

The relationship between incidence and intensity of flares-ups and the vitality of the treated teeth has been investigated, yet with conflicting results. Mor et al. [7] found that flare-ups more often followed endodontic treatment in nonvital teeth and after retreatment than in vital teeth. However, Harrison et al. [8, 9] reported that the incidence and intensity of flare-up were unrelated to tooth vitality. No correlation has been found between pulp status and any PEP [10, 11].

PEP (not limited to flare-up) is very frequent after endodontic treatment, and more than 50% of those who feel any PEP experienced severe pain [3]. Nevertheless, no study has evaluated the incidence and severity of PEP after re-treatment and after initial RCT of teeth with vital or necrotic pulp [3].

The purpose of this study was to evaluate the incidence, severity, and types of PEP presenting after root canal treatment in teeth with vital or necrotic pulp and after retreatment.

## 2. Materials and Methods

### 2.1. Study Population

This is a prospective study of individuals who underwent RCT in teeth with vital pulp, necrotic pulp, or vital pulp that had been treated for symptomatic irreversible pulpitis, or who received retreatment of the root canal, by one endodontic clinician during an eight-month period

A structured questionnaire accessed age, gender, tooth location, and pulpal diagnosis (vital pulp, previously initiated therapy, or necrosis). The Ethics Committee of Tel Aviv University approved the study, and all patients signed informed consent.

### 2.2. Inclusion Criteria

Inclusion criteria were treatment of only one tooth, completion of treatment in one session, and the absence of preoperative pain (otherwise the treatments were preformed in two sessions). Indications for treatment were (1) teeth with vital healthy pulp that were treated for prosthetic reasons. These teeth were treated by the endodontic practitioner only (BB); (2) teeth with previously initiated therapy consequent to symptomatic irreversible pulpitis, which were dressed with anti-inflammatory medicine (Ledermix paste, Haupt Pharma GmbH, Wolfratshausen, Germany); (3) teeth with necrotic pulps (diagnosed by a negative response to cold stimulation and an absence of blood on entry to the root canal), with or without apical periodontitis as evidenced by a periapical radiograph, but without preoperative pain; (4) teeth that were designated for endodontic retreatment due to apical periodontitis or prosthetic reasons, but without preoperative pain.

### 2.3. Exclusion Criteria

Exclusion criteria were the presence of teeth with symptomatic irreversible pulpitis, preoperative pain, or necrotic pulp associated with clinical symptoms such as swelling or purulence. In addition, patients who were being treated with antibiotics were also excluded from the present study.

### 2.4. Operative Endodontic Treatment

Maxillary teeth were anaesthetized before treatment by infiltration and mandibular teeth by mandibular alveolar nerve block, using one cartridge of Lidocaine 2% with 1 : 100,000 epinephrine or Mepivacaine 3% (in patients for whom epinephrine was contraindicated), using 27 gauge needles. Local anesthesia was delivered to all teeth that were treated or that were candidates for retreatment of root canals, to prevent the evocation of pain from pressure of rubber dam clamps on the gingiva or from over instrumentation, leakage of root canal irritants, or overfilling material.

 In all operative procedures, a rubber dam was applied immediately after delivery of local anesthesia. The endodontic treatment included accessing the root canal(s), hand instrumentation for extirpation, debridement, and shaping the canals, as necessary. In retreatment, the gutta-percha was dissolved by xylene. The working length was determined by Root ZX apex locator (J. Morita, California, USA). Canals were irrigated with 5 mL of 3.5% NaOCl and sterile saline and obturated with laterally condensed gutta-percha and AH26 sealer (the obturation length was determined by the working length and was 0.5–1 mm short of the radiographic apex). The duration of treatment ranged between 45 and 60 minutes.

### 2.5. Determination of Pulp Status

The pulp status was determined and recorded as vital only when the tooth responded immediately before treatment to a cold stimulus (CO_2_ snow) and/or there was evidence of haemorrhage on opening the pulp chamber. The pulp status was recorded as nonvital if there was no response to cold and no evidence of haemorrhage on opening. Periapical pathology status was determined by a periapical radiographic evaluation.

### 2.6. Evaluation of Postendodontic Pain and Use of Analgesic Drugs 24 h Postoperatively

The treating dentist (BB) informed the patients that PEP may develop and suggested they take Acetaminophen to relieve severe pain. A student (MG), unaware of the treatments performed, telephoned patients within 24 h postoperatively. She asked them to grade the level of pain they felt 6 and 18 h after treatment, using a continuous 1–5 point scale (1: no pain, 2: mild pain, 3: moderate pain, 4: severe pain and 5: very severe/unbearable pain), which they had seen when they signed the consent form. Patients were also asked to specify the type of pain from which they suffered (spontaneous or stimulated by mastication or palpation). Additional explanations about the scale were provided by the student, as necessary, until clarity was reached. Patients were asked about their use of analgesic drugs following the treatment.

### 2.7. Statistical Analysis

The independent student's *t*-test and one- or two-way variance test were used to compare the continuous variables between groups. Chi-square was used to compare frequencies of categorical variables. Differences were considered significant when probabilities were less than 0.05.

## 3. Results

### 3.1. Patients and Treated Teeth

During the study period, 274 individuals met inclusion and exclusion criteria. All patients responded to the questionnaire (100% response rate). The distribution of patients according to age, gender, and pulp condition is presented in Table 1. Treated teeth comprised 97 (35.4%) anterior teeth, 89 (32.5%) maxillary molars, and 88 (32.1%) mandibular molars.

### 3.2. Incidence and Intensity of Postendodontic Treatment Pain

**Six h after Treatment**

The mean incidence of PEP was 54.7% (150/274). No pain (degree 1) was reported in 45.3% of the patients (124/274). A low level (degree 2) was reported in 17.5% (48/274), moderate level in 20.4% (55/274), and a high level (degrees 4 and 5) in 17.1% (47/274).

**18 h after Treatment**

The mean incidence of PEP was 46.4% (127/274). No pain (degree 1) was reported in 53.6% (147/274). A low level of pain (degree 2) was reported in 22.3% (61/274), a moderate level in 13.9% (36/274), and a high level (degrees 4 and 5) in 10.2% (28/274).

### 3.3. Effect of Pulp Condition on PEP and Analgesic Use

 Six hours posttreatment, incidence and intensity of PEP were higher among patients who received RCT in teeth with vital pulp than in teeth with necrotic pulp or retreated teeth (Table 2). No such correlation was found 18 h after treatment (Table 2). The type of endodontic treatment was not found to be correlated with the frequency of analgesic use or with the level of pain relief following the use of an analgesic.

### 3.4. Effect of Pulp Condition on the Type of PEP: Spontaneous or Stimulated Pain

 No statistical relation was found between the pulp condition and the type of pain (stimulated or spontaneous) 6 or 18 h after treatment (Table 3).

### 3.5. Effect of Gender on PEP

 Gender was significantly associated with the intensity of PEP. After treatment, women reported a higher mean pain intensity than men, 6 h (2.29 ± 1.38 (SD) versus 1.95 ± 1.19 (SD), resp., *P* < 0.034) and 18 h (1.97 ± 1.21 (SD) versus 1.68 ± 0.99 (SD), resp., *P* < 0.041).

### 3.6. Effect of Tooth Location on PEP

 There was no statistically significant correlation between tooth location and the intensity of PEP, 6 and 18 h after treatment.

## 4. Discussion

In the present study the incidence of PEP was high, ranging from 34.6% to 63.8%, depending on the pulp condition. RCT of teeth with vital pulp was associated with a higher incidence and intensity of PEP (6 h after treatment) compared to teeth with necrotic pulp or retreated teeth. This is in accordance with Levin et al. [3] who showed that 53% of patients receiving root canal treatment reported PEP; of them, only 21% reported a low level of pain. In contrast, other studies showed lower frequency even for single appointment groups [6, 12, 13]. However, those studies included only patients with flare-up; in the present study we included all patients who reported any level of PEP.

 Another factor that may contribute to the higher frequency of PEP in the present study is that root canal treatment was performed at a single visit. Single-visit treatment has been shown to result in higher frequency of PEP, and consequently higher consumption of analgesics [6, 10, 13–15]. Nevertheless, the main advantages of single visit treatment are the reduced time and added convenience for both patient and dentist, without increasing short or long complications [14].

 Evidence in the literature of the effect of pulp status (vital or necrotic) on the incidence and severity of PEP is inconclusive. Our findings concur with those of Clem [16] and Calhoun and Landers [17], Marshal and Liesinger [11], Fox et al. [18], and Undoye and Jafarzadeh [19], who found that PEP is more common following treatment of teeth with vital pulp.

 In contrast, Albashaireh and Alnegrish [20], Mor et al. [7] and Mattscheck et al. [21] reported greater incidence of PEP following treatment of teeth with necrotic pulps. The discrepancy may be due to different criteria used to evaluate PEP or to different endodontic materials and techniques. The findings of the present study also contrast with those of previous studies that reported statistically significant correlations between the presence of periapical lesions and rates of flare-ups after root canal treatments that were performed by students or residents [22, 23]. Treatment by students or residents may be a reason for the discrepancy here, in addition to the fact that those studies evaluated only patients with flare-up.

 The reason for the higher incidence and severity of PEP after treatment of teeth with vital pulp is not completely clear. One possibility is that the injury of periapical vital tissue during endodontic treatment in teeth with vital pulp promotes more intensive secretion of inflammatory mediators, such as prostaglandins, leukotrienes, serotonin, histamine, and bradykinin (all of which are also pain mediators).

 Here we reported significantly higher levels of PEP after initial RCT (of teeth with vital pulp) than after retreatment. This contrasts with the study conducted by Mattscheck et al. [21] in which no difference was observed between pain after initial root canal treatment and after retreatment. The difference between these two studies may be attributed to the different populations, culture, and attitude to pain, different pathology between teeth in the retreatment group, and different treatment and obturation materials and techniques.

 In the present study, teeth with symptomatic irreversible pulpitis were treated previously by general practitioners who placed Ledermix at the pulp exposure site to relieve dental pain. The effect of anti-inflammatory agents on the pain of such teeth has been investigated previously. Moskow et al. [24] reported a statistically significant reduction in the incidence of pain 24 h postoperatively, following placement of corticosteroid as an intracanal anodyne.

 Higher levels of PEP among women in the current study concur with investigations by Albashaireh and Alnegrish [20], Torabinejad et al. [25], Ng et al. [26], Al Bashaireh and AlNegrish [20], and Al-Negrish and Habahbeh [12]. Differences between the genders may be explained by differences in physiological reaction to pain or by less reporting by men, due to societal expectations that they tolerate pain more than women [27].

 Attention to differences, according to pulp status, in the prevalence and severity of pain following endodontic treatment, may guide clinicians in informing patients about expected pain and in prescribing analgesics for use immediately after treatment. Management of pain should be an integral part of dental treatment, particularly in its initial stages, to prevent exacerbation. The final decision for prescribing an analgesic should consider such variables as gender, number of treatment sessions, and a patient's past experience with pain and with analgesics.

## 5. Conclusion

 Root canal treatment of teeth with vital pulp induced a significantly higher incidence and intensity of PEP than did treatment of teeth with necrotic pulp or retreated teeth. Dentists should be aware of this pain and make efforts to prevent or treat it. Patients should be informed about the possibility of pain after endodontic treatment and instructed in the use of analgesics.

## Figures and Tables

**Table 1:** Patient distribution according to gender, age, and treated teeth for each of the treatment groups.

Treatment groups	Number of patients* (%)	Gender M/F	Age (Y)	Tooth type (anterior/max molar/mand molar*)
Vital pulp	141 (51.5)	52/89	50.9	36/51/54
Necrotic pulp	52 (19)	24/28	56.4	31/11/10
Retreatment	81 (29.6)	26/55	45.1	30/27/24

*max molar: maxillary molar; mand molar: mandibular molar.

**Table 2:** Incidence and intensity of post-endodontic pain (PEP) (Scale 1–5), 6 and 18 h after treatment.

	6 hours		18 hours	
Treatment groups	Incidence number (%)	Intensity mean ± SD	Incidence number (%)	Intensity mean ± SD
Vital pulp	90 (63.8)	2.46 ± 1.4	73 (51.8)	2.00 ± 1.2
Necrotic pulp	20 (38.5)	1.78 ± 1.2	18 (34.6)	1.56 ± 0.9
Retreatment	40 (49.4)	1.89 ± 1.1	36 (44.4)	1.81 ± 1.1
*P* value	0.003	0.001	NS	NS

**Table 3:** Distribution of type of postoperative pain (PEP) after 6 and 18 hours in relation to the different treatment groups.

Treatment groups	6 hours after treatment			18 hours after treatment		
	Number of patients	Type of PEP		Number of patients	Type of PEP	
		Spontaneous number (%)	Stimulated number (%)		Spontaneous number (%)	Stimulated number (%)
Vital pulp	90	73 (81.1)	17 (18.9)	74	33 (44.6)	41 (55.4)
Necrotic pulp	20	17 (85)	3 (15)	18	6 (33.3)	12 (66.7)
Retreatment	40	32 (80)	8 (20)	37	22 (59.5)	15 (40.5)
